# Supplementary material for: The Antioxidant Power of Bergamot Polyphenolic Fraction Gold Potentiates the Effects of L-Citrulline in Athlete Performance and Vasodilation in a Pilot Study
Source: Nutrients. 2025 Mar 21;17(7):1106. doi: 10.3390/nu17071106 (PMC11990273; doi:10.3390/nu17071106)
Supplement: Supplementary file 1 [file nutrients-17-01106-s001.zip › nutrients-3530691-supplementary.pdf]

BPFG Chromatogram

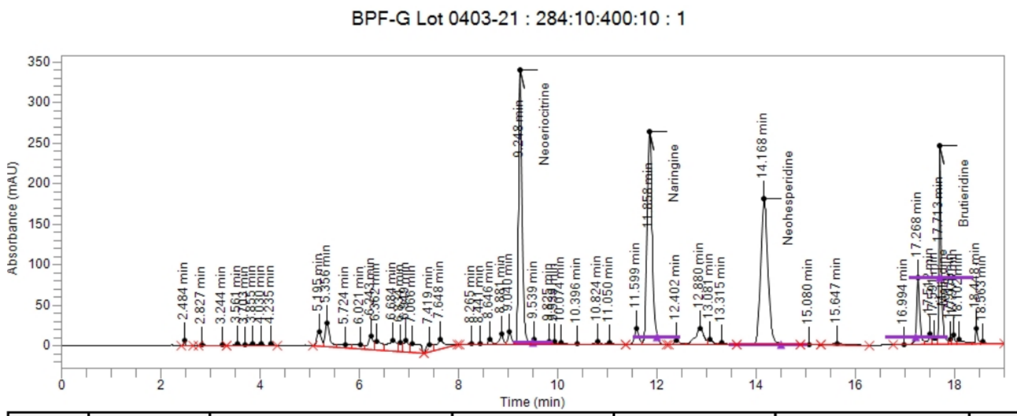

Peak #	Time	Component Name	Area	Height	Final Amount	Units
47	17.713	Brutieridine	656,827.3	246,131.4	56,496.8320	µg/g
44	17.268	Melitidine	349,250.1	83,830.6	27,950.9345	µg/g
35	11.858	Naringine	1,874,484.3	263,414.2	138,945.5648	µg/g
26	9.248	Neoeriocitrine	1,618,264.9	340,179.9	131,942.1912	µg/g
40	14.168	Neohesperidine	1,838,350.9	180,787.0	134,617.2130	µg/g
**Total**			6,337,177.4		489,952.7356	

The phytochemical characterization of BPFG performed by HPLC showed the presence of more than 47% of bioactive compounds. As shown by the chromatogram BPFG was abundant in Brutieridine (5.6%),Melitidine (2.7%), Naringine (13.8%), Neoeriocitrine (13.1%) and Neohesperidine (13.4%).

Bergamot Polyphenolic Fraction Gold® (BPFG®)

SPECIFICATION SHEET

**ID Code**	**BRGESTPG**	
**Trade name**	**BERGAMOT POLYPHENOLIC FRACTION GOLD® (BPFG®)**	
**DESCRIPTION**	**SPECIFICATIONS**	
Botanical Source	Citrus Bergamia Risso et Poit.	
Family	Rutaceae	
Synonyms	Citrus aurantium var. bergamia	
Country of Origin	Calabria, Italy	
Part Used	Fruit Juice	
Shelf Life	4 years, if correctly stored	
**ORGANOLEPTIC CHARACTERISTICS**	**SPECIFICATIONS**	
Colour	Yellow Powder	visual (CQ-MO-148)
Odour	Aromatic	visual (CQ-MO-148)
Flavour	Characteristic of bergamot	sensory (CQ-MO-148)
**CHEMICAL CHARACTERISTICS**	**SPECIFICATIONS**	**METHOD**
pH	3.0 – 4.0	IM (0.5% in water at 25°C)
Average Mesh Size	Pass 60 mesh	Sieve: (CQ-MO-023)
Bulk Density	40 - 60 g/100mL	PT CHIM 65 rev 0 2011
Moisture Content	< 8.0%	ISTISAN 96/34, pag 7
Solubility in 40°C H_2O	Good	visual: (CQ-MO-148)
Solubility in 50% H_2O + EtOH	Good	visual: (CQ-MO-148)
Organic Solvent Residue	Complies with Dir. 2009/32/CE	GC: (CQ-MO-168)
Pesticides Residue	Complies with Reg. UE 839/2008	PT CHIM 69rev 02 011
Tot. active ingredient strength (Neoeriocitrin, Naringin, Neohesperedin, Melitidin, Brutieridin)	> 47.0 %	HPLC
**SINGLE COMPONENT MINIMUM TRESHOLD CONCENTRATION**		
Neoeriocitrin	9.0 %	
Naringin	11.0 %	
Neohesperidin	11.0 %	
Melitidin	1.0 %	
Brutieridin	2.0 %	
**HEAVY METALS**	**UNIT**	**RANGE**
Arsenic	mg/kg	<1.0
Lead	mg/kg	<0.5
Cadmium	mg/kg	<0.5
Mercury	mg/kg	<0.3
**MICROBIOLOGICAL EVALUATION**		
Aerobic Plate Count	<1,000 CFU/g	ISO 4833-1:2013
Yeast and Mold Count	<100 CFU/g	ISO 21527-1:2008
E. Coli	Negative	ISO 16694-2:2001
Coliform	Negative	ISO 4832:2006
Salmonella	Negative	UNI EN ISO 6579:2000
Staphylococcus Aureus	Negative	UNI EN ISO 6888-2:2004
Streptococci	Negative	PT BAT26 rev0 02012
**PRODUCT TREATMENT**		
Drying Method	**Spray dry**	

Herbal &Antioxidant Derivatives S.r.l. (H&AD S.r.l.)
Headquarters: **Località Chiusi - 89032 BIANCO (RC), ITALY**
Tel. +39 0964 913405 E-mail: info@head-sa.com *Website:* *http://www.head-sa.com/*
CF/PI- 02543180802

NUTRITIONAL FACTS

**PRODUCT NAME:**	**BPFG®**
**IDENTIFICATION CODE:**	**BRGESTPG**
**NUTRIENTS**	**AMOUNT per 100g**
Calorific Value (kcal)	**300 - 450**
Fat (%)	**< 1.0**
Ash (%)	**< 7.0**
Total Carbohydrates (%)	**> 80.0**
Protein (%)	**< 2.5**
Water (%)	**< 8.0**

RECOMMENDED STORAGE CONDITIONS:

Store in a fresh and dry place, repaired from light and humidity sources.

BEST BEFORE:

48 months under the previously mentioned conditions and in its original packaging.

PACKAGING:

20 kg fiber drums or 10 kg Aluminium bags.

Giuseppe Trunfio

H&ad srl analytical chemistry & processes

***E-mail :* g.trunfio@head-sa.com**
